# Comparison of microleakage under orthodontic brackets bonded with five different adhesive systems: in vitro study

**DOI:** 10.1186/s12903-023-03368-2

**Published:** 2023-09-05

**Authors:** Nela Masarykova, Emil Tkadlec, Zdenek Chlup, Jan Vrbsky, Alena Brysova, Pavlina Cernochova, Lydie Izakovicova Holla

**Affiliations:** 1 Clinic of Stomatology, Institution Shared with St. Anne’s University Hospital, Faculty of Medicine, Masaryk University, Pekařská 53, Brno, 656 91 Czech Republic; 2 Faculty of Science, Palacky University Olomouc, 17. listopadu 1192/12, Olomouc, 779 00 Czech Republic; 3 Institute of Physics of Materials of the Czech Academy of Sciences, Žižkova 513/22, Brno, 616 00 Czech Republic; 4 International Clinical Research Center (ICRC), St. Anne’s University Hospital, Pekařská 53, Brno, 656 91 Czech Republic

**Keywords:** Adhesive, Bracket, Demineralization, Microleakage, Orthodontics, Thermal cycling

## Abstract

**Background:**

Orthodontic treatment is associated with numerous adverse side effects, such as enamel discoloration, demineralization or even caries. The presence of microleakage between the enamel and the adhesive and between the adhesive and the base of the orthodontic bracket allows penetration of the bacteria, molecules, and liquids into the enamel and can lead to unpleasant “white spot lesions” or secondary caries beneath and around the brackets. The aim of this in vitro study was to evaluate microleakage in five adhesive systems commonly used in orthodontic practice for bonding brackets.

**Methods:**

One hundred extracted premolars were divided into five groups of twenty teeth. Stainless steel Legend medium metal brackets were bonded to teeth using five adhesive systems: resin-reinforced glass ionomer cement GC Fuji Ortho LC (GCF) and composite materials Light Bond (LB), Transbond XT (TB), Trulock™ Light Activated Adhesive (TL), and GC Ortho Connect (GCO). The specimens were subjected to thermal cycling, stained with 2% methylene blue, sectioned with low–speed diamond saw Isomet and evaluated under a digital microscope. Microleakage was detected at the enamel-adhesive and adhesive-bracket interfaces from occlusal and gingival margins. Statistical analysis was performed using generalized linear mixed models with beta error distribution.

**Results:**

Microleakage was observed in all materials, with GCF showing the highest amount of microleakage. Composite materials GCO, TB, and LB exhibited the lowest amount of microleakage with no statistical difference between them, while TL showed a statistically significantly higher amount of microleakage (p < 0.001). The enamel–adhesive interface had more microleakage in all composite materials (GCO, LB, TB, and TL) than the adhesive bracket–interface (p < 0.001). The highest amount of microleakage occurred in the gingival region in all materials.

**Conclusion:**

Composite materials showed better adhesive properties than a resin-reinforced glass ionomer cement. The presence of microleakage at the enamel-adhesive interface facilitates the penetration of various substances into enamel surfaces, causing enamel demineralization and the development of dental caries.

## Introduction

Patients who undergo orthodontic therapy with fixed orthodontic appliances face a challenging oral hygiene situation, which can be associated with adverse side effects such as enamel discoloration, demineralization (white spot lesions) or even caries [1–3]. The reported prevalence of enamel demineralization varies from 33.8 to 97% [4, 5]. Although in some cases, these demineralization lesions may be reversible, the chalky appearance of the enamel surface can be partially neutralised by salivary proteins that remineralize the enamel surface [6], in the case of orthodontic treatment, these lesions frequently evolve progressively and become irreversible, leading to caries processes [7]. The main reason is higher retention of plaque due to the presence of orthodontic brackets, bands, and other devices. However, etching the enamel and the type of sealants and composite resins used for bonding the brackets also play an important role. In this context, microleakage under orthodontic brackets is of considerable clinical importance. Microleakage may be defined as the passage of bacteria, fluids, molecules or ions between the adhesive material and the enamel or between the adhesive material and the base of the orthodontic bracket, which can initiate enamel demineralization and development of caries or lead to debonding the bracket, thus prolonging the duration of the orthodontic therapy [8].

Nowadays, the most used adhesive systems for bonding of orthodontic brackets are resin-based composite materials that harden through the process of radical polymerization and resin-reinforced glass ionomer cements. Composite material is a mixture of two materials that behave as a single one. It is composed of organic (resin, the coupling agent and the initiator) and inorganic components (the filler – aluminium oxide, silicon dioxide and phosphate) [9, 10]. Resin increases mechanical and aesthetic properties and adhesion to the dental enamel. Bisphenol A glycerolate dimethacrylate (Bis-GMA), urethane-dimethacrylate monomer (UDMA), and triethylene glycol dimethacrylate (TEGDMA) are commonly used resin monomers in dentistry [11]. Functional monomers like 10-methacryloyloxydecyl dihydrogen phosphate (10-MDP), a compound of GC Ortho Connect, interact with hydroxyapatite and create a chemical bond, which has been proven to be stable in aqueous environment [12, 13]. Resin-reinforced glass ionomer cements were developed to improve the parameters of conventionally used glass ionomer cements. It is a combination of composite resin 2-hydroxyethyl methacrylate (HEMA) and fluorosilicate glass that increase adhesion to the dental enamel and improve its mechanical properties [9, 14].

Microleakage in adhesive materials may be caused by many factors, such as polymerization shrinkage, inadequate adhesion, and in the case of resin-reinforced glass ionomer cement, the disturbance of water balance during the solidification process [15]. The process of curing of composite materials consists of three phases: pre-gel, gel-point, and post-gel. During the pre-gel phase, the molecules rearrange inside the material [16]. We assume that the contractile force may result in the gap formation or leakage development between the enamel and adhesive, or between the adhesive and the base of the orthodontic bracket [16, 17]. During the post-gel phase, polymerization shrinkage occurs as the material loses its flow ability and the generated force is transmitted to the composite interface [18, 19]. Thermal changes and mechanical forces also play an important role in the oral cavity. Thermal changes caused by hot and cold dishes and beverages lead to the expansion and contraction of materials in the mouth. On average, the oral cavity experiences around ten thermal cycles per day. The average temperature in the oral cavity is about 35 °C [20, 21], with the lowest possible temperature at the tooth surface being around 0 °C [22] and the highest between 55 and 60 °C [17, 20, 21, 23]. These processes can lead to the development of microleakage underneath the orthodontic brackets or debonding from the metal or dental surface [24]. On the contrary, the total-etch process increases adhesive properties of these materials. The mechanism is based on enamel deproteinization and formation of microporosities for the micro-mechanical interlocking of the resin monomers into spaces created in enamel [25, 26].

Various methods have been employed to evaluate microleakage around brackets in vivo and *in vitro.* Although clinical relevance of in vitro leakage tests does not always correlate with the current clinical situation, these tests are the most frequently used laboratory examinations to study mechanisms of fluid leakage. In vitro studies include the use of tracers such as dyes, radioactive isotopes, chemical tracers, and bacteria, as well as marginal percolation of water and subjection to air pressure, neutron activation analysis (NAA), scanning electron microscopy (SEM), and electrical conductivity [27].

The aim of this in vitro study was to evaluate the development of microleakage under the metal orthodontic brackets in four composite materials and resin reinforced glass ionomer cement commonly used in orthodontics for bonding brackets. As a novelty, we tested the composite material Trulock™ Light Activated Adhesive in which microleakage occurrence has not been tested yet. The null hypothesis of this study states that type of adhesive system does not affect the amount of microleakage under orthodontic brackets.

## Materials and methods

The specimens were 100 premolars extracted for orthodontic reasons from 45 patients between 13 and 40 years of age treated at the Orthodontic Department of the Clinic of Stomatology in St. Anne’s University Hospital in Brno, the Czech Republic. The informed consent was received from all participants. The Ethics Committee of St. Anne’s University Hospital in Brno approved the study with reference number EK – FNUSA-29/2022. Inclusion criteria were non-carious premolars from the upper or lower jaw. Exclusion criteria included carious, filled, endodontically treated, or damaged teeth, and teeth with already bonded brackets or attachments. The premolars were cleaned and soaked in 0.5% chloramine T solution for 24 h to prevent bacterial growth, then stored in physiological solutions for up to four months. The teeth were then dried with oil and moisture-free compressed air. Legend medium metal brackets (GC Orthodontics America, Alsip, IL 60,803, USA) were bonded to the enamel of the premolars using either resin-reinforced glass ionomer cement or one of four composite materials. The brackets were bonded using the direct bonding method.

One hundred premolars were divided into five groups per twenty teeth. The five adhesive materials included Transbond XT (3 M, Unitek TM, Monrovia, CA, USA), + orthophosphoric acid + liquid primer, composite material, Light Bond (Reliance Orthodontic Products, Inc., Itasca, USA), composite material, Trulock™ Light Activated Adhesive (RMO, INC., Denver, USA), composite material, GC OrthoConnect (GC Corporation, Tokyo, Japan), composite material, and GC Fuji Ortho LC resin reinforced glass ionomer cement (GC Corporation, Tokyo, Japan).

Photopolymerization was performed using the LED Ortholux ^TM^ Luminous Curing Light. After bonding the orthodontic brackets, the specimens were stored in a physiological solution and subjected to thermal cycling. Thermocycling protocol was established to simulate two months of orthodontic therapy (six hundred cycles). The specimens were immersed in 2.5 °C (± 2 °C) and 56 °C (± 2 °C) baths for 5 min and 2 min 15 s, respectively, the teeth were stored at room temperature. One cycle lasted 14 min 30 s. Total thermocycling took 145 h (6 days).

**Fig. 1:**
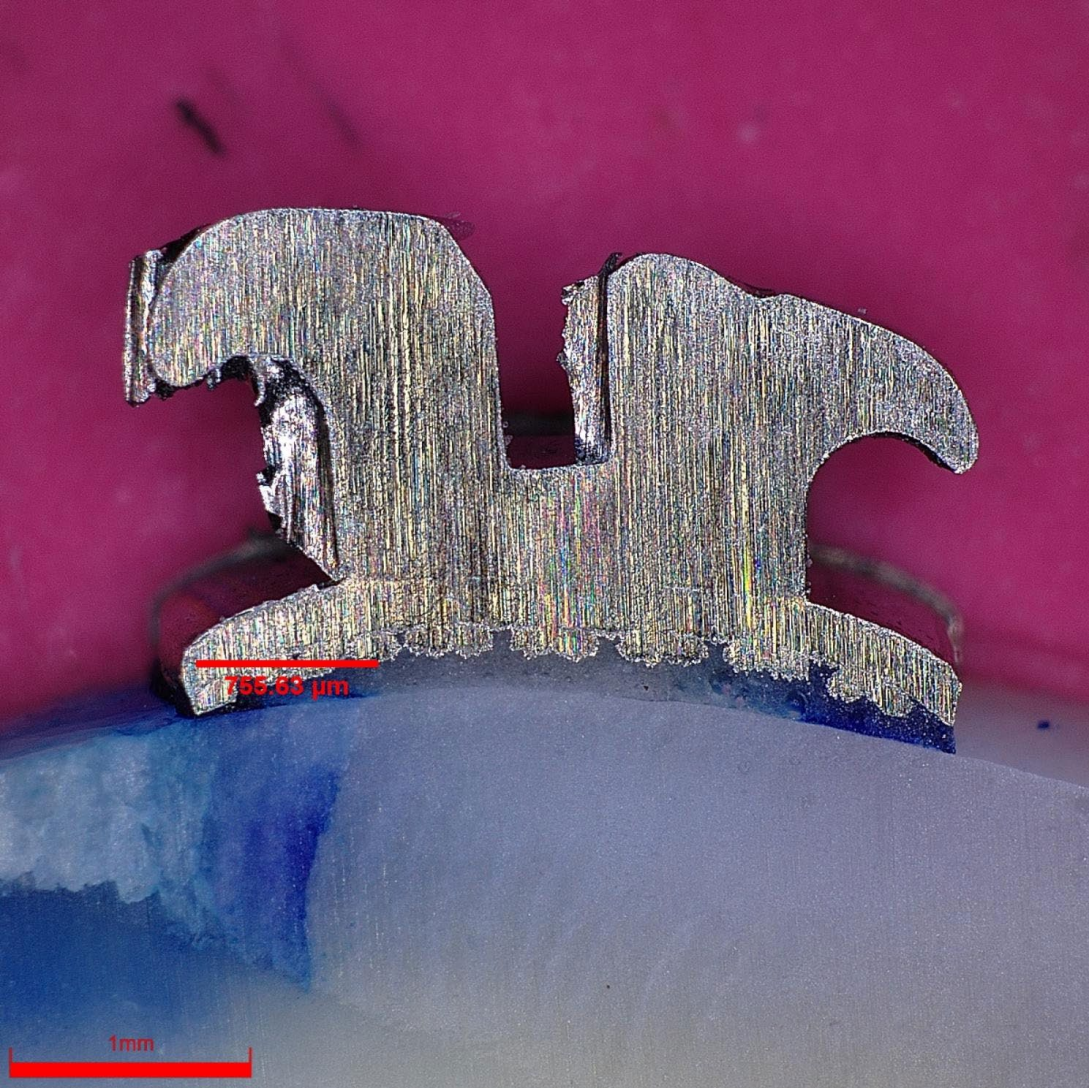
A magnified view (60×) of a section of a metal orthodontic bracket (Legend medium metal bracket by GC Orthodontics) with methylene blue staining indicating microleakage between the adhesive material Transbond XT (by 3 M Unitek) and enamel. The image was captured using a digital microscope (OLYMPUS DSX 1000)

All samples were covered with nail varnish, leaving 1 mm around the bracket margins and then dyed with 2% methylene blue solution. The apexes of the samples were embedded in resin blocks and three longitudinal sections were made using a diamond saw Isomet. The samples were examined under a digital microscope Olympus DSX 1000 with 60 × magnification (Fig. 1). Methylene blue penetration was measured at the enamel–adhesive and adhesive–bracket interfaces from both occlusal and gingival margins. Programme Image J was used to measure the extent of methylene blue penetration. The measurement was repeated by the same examiner after two weeks.

A total of 2400 measurements were initially obtained, but some failed cuts and measurements were discarded, leaving 2333 measurements for statistical analysis: GC Fuji Ortho (GC): *n* = 470, Light Bond (LB): *n* = 480, Transbond XT (TB): *n* = 448, Trulock™ Light Activated Adhesive (TL): *n* = 463, GC Ortho Connect: *n* = 472.

### Statistical analysis

The extent of microleakage was measured as the sum of the penetrations from both the occlusal and gingival margins of the bracket. Since the total length of the bracket is 3 mm, the measurements are bounded between 0 and 3 mm. Dividing by three converts the data to proportional dye penetration (PDP) ranging from 0 to 1. This variable was used in statistical models as a response variable. From a statistical point of view, the proportions thus obtained are continuous proportions that differ from ordinary proportions arising as ratios of the observed and total counts whose statistical distribution follows the binomial distribution. Consequently, we modelled the variation in PDP by using generalized linear mixed models (GLMM), when the error distribution fitted the beta distribution [28]. We used a logit transformation as a link function. As fixed effects, two categorical variables were included in the model structures: adhesive (5 levels: GC, LB, TB TL, GCO) and measurement position with two levels (under the bracket or the adhesive). Because the repeated measurements on one tooth are not independent similarly to those made on one cut, we incorporated a hierarchical random effect id/r, i.e., cut within the identity of a tooth (100 teeth in total). The variable cut had three levels (cut 1, cut 2, and cut 3). Since the beta distribution does not include 0 and 1, these values were replaced by 0.01 and 0.99. This approach assumes that the measured 0 and 1 s did not arise from any process other than the values within this interval. All calculations were performed using the glmmTMB package in the R program [29, 30]. We built a set of five statistical models that included one without predictors (intercept-only model), one with adhesive, one with position, one with adhesive and position, and finally one with adhesive, position, and interactions between adhesive and position. First, we focused on the differences in penetration between the tested adhesives and then we incorporated position into the model structure. We used the lowest value of Akaike’s information criterion (AICc) [31] to identify the model most supported by data. The difference in AICc greater than 2 was considered strong evidence for the best model. The emmeans package [32] was used to calculate multiple means for individual predictor levels, their 95% confidence limits, and their statistical comparison. Post-hoc multiple comparisons between means were performed according to Tukey’s method.

Furthermore, to test the effect of the tooth side (occlusal or gingival) on dye penetration, we removed all rows where the dye had completely penetrated (PDP = 1) from the data set. This reduced the size of the dataset to *n* = 3681. Then we added a two-level variable Side (gingival/occlusal) to the GLMM structure and created a set of models with three predictors.

The influence of the variable Side was evaluated in the same way as described above.

## Results

**Fig. 2:**
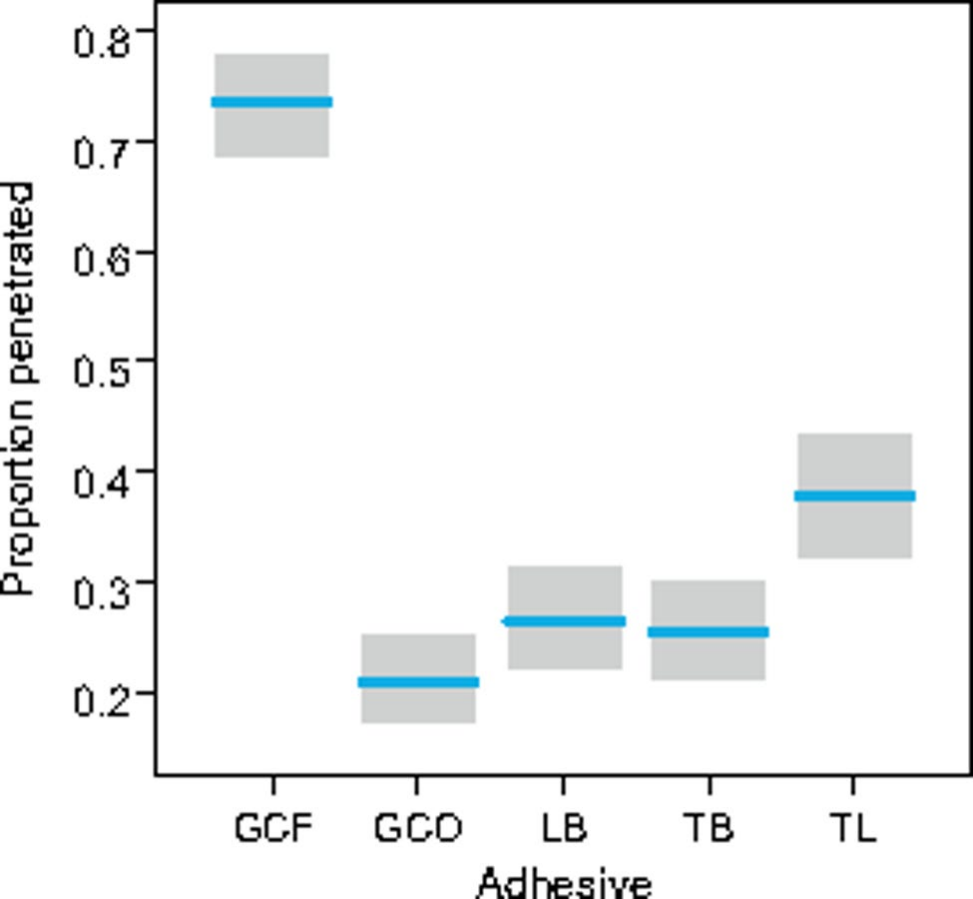
Proportional dye penetration (PDP) for five adhesive systems (resin-based composite materials: GC – GC OrthoConnect, LB – Light Bond, TB – Transbond XT, TL – TrulockTM Light and resin-reinforced glass ionomer cement: GCO – GC Fuji Ortho LC) as predicted by a generalized linear mixed model containing the adhesive as a predictor. PDP is measured as the sum of proportions from both the occlusal and gingival margins of the bracket. The shaded areas indicate 95% confidence intervals

Methylene blue PDP was present in all adhesive materials. The average PDP predicted by the model containing adhesive as a predictor was 0.402 mm (95% CI 0.372–0.434–1.206), which corresponds to 1.21 mm (1.116–1.302) of the total bracket length of 3 mm. However, considerable differences were found among the adhesives used (Fig. 2).

The greatest PDP of 0.733 (95% CI 0.685–0.776), corresponding to the total bracket length of 2.199 mm (2.040–2.343), was observed in resin-reinforced glass ionomer cement GC Fuji Ortho. The PDP for GC Fuji Ortho was incomparably higher than in other adhesive materials (GC–LB: p < 0.001, GC–TB: p < 0.001, GC-TL: p < 0.001, GC – GCO: p < 0.001). The second highest PDP of 0.377 (0.323–0.432) corresponding to 1.131 mm (0.957–1.317) of the total bracket length was observed in the composite material Trulock™ Light Activated Adhesive. The PDP was proved to be higher than in the remaining three composite materials (TL–LB: p = 0.02, TL–TB: p = 0.010, TL – GCO: p < 0.001). No differences were found among the composite materials Light Bond, Transbond XT and GC Ortho Connect (Transbond XT: PDP = 0.255 (0.209–0.306), length = 0.765 mm (0.627–0.918); Light Bond: PDP = 0.265 (0.218–0.318), length = 0.765 mm (0.654–0.954); GC Ortho Connect: PDP = 0.208 (0.172–0.250), length = 0.624 mm (0.516–0.75); differences: LB–TB: p = 0.99, TB – GCO: p = 0.52, LB – GCO: p = 0.33).

**Fig. 3:**
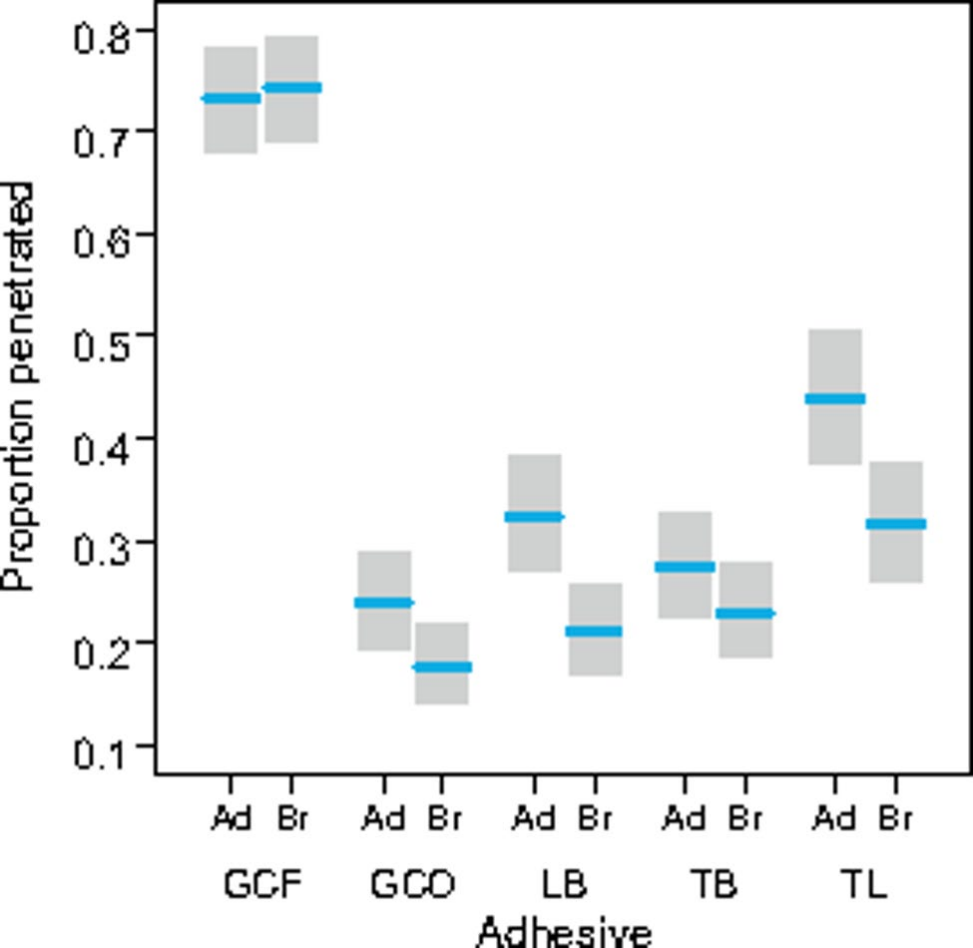
Proportional dye penetration (PDP) for adhesive–enamel (Ad) and adhesive–bracket interface (Br) of five adhesive materials (resin-based composite materials: GC – GC OrthoConnect, LB – Light Bond, TB – Transbond XT, TL – TrulockTM Light and resin-reinforced glass ionomer cement: GCO – GC Fuji Ortho LC) as predicted by a generalized linear mixed model containing the adhesive materials, position of measurement and the interaction between adhesive and position as predictors. The shaded areas indicate 95% confidence intervals

The model with predictors adhesive and position of measurement and interaction adhesive*position was the best supported (difference in AICc = 16.5). The presence of interaction term in the model suggests that the effects of both factors on dye penetration were not additive and changed depending on the adhesive or position (Fig. 3). This model demonstrates not only the differences between the adhesives used but also the differences in dye penetration under the bracket and the adhesive. While the differences in position are small with the GC adhesive and even larger under the bracket, the penetrations are greater under the adhesive than under the bracket for the other adhesives. Data analysis on the reduced dataset showed that dye penetrations are 1.5 times deeper on the gingival side of the tooth (the AICc drop after adding the Side variable to the model is 170).

## Discussion

Microleakage occurs in adhesive systems when fluids from the oral cavity penetrate underneath orthodontic brackets, leading to the formation of decalcified, white spot lesions on the enamel [33, 34] or debonding the bracket and thus prolonging the orthodontic therapy [8]. This in vitro study was designed to investigate the amount and location of microleakage in five selected adhesive systems commonly used in orthodontic practice. The null hypothesis of the study was rejected as there were differences in the microleakage between different composite materials and resin-reinforced glass ionomer cement.

All adhesive materials assessed showed some degree of microleakage. Some authors confirmed that the occurrence of microleakage does not depend on the type of adhesive materials used [35, 36]. We found the largest amount of microleakage in the resin-modified glass ionomer cement GC Fuji Ortho LC, which has also been reported by other authors [35, 37]. The higher occurrence of microleakage in this material compared to the selected composite materials can be attributed to the chemical composition, which makes the material more porous. In addition, violation of the procedure of mixing powder and liquid or over-drying of the enamel surface may also contribute to higher microleakage. However, the exact explanation for the higher presence of microleakage in resin-reinforced glass ionomer cement is not clear yet and should be further investigated.

In contrast, GC Ortho Connect, Light Bond and Transbond XT composite materials showed the lowest microleakage, the amounts did not differ. These materials have very similar chemical properties. Transbond XT was the most tested material in previous studies. Some authors detected that Transbond XT exhibited lower microleakage than other tested composite materials [8, 35, 38]. On the other hand, Buyuk et al. [39] reported even lower microleakage for low-shrinking composite Filtek™ Silorane as well as Vicento et al. [40] who reported lower microleakage for flowable giomer (Beautiful - Flow). Hedayati et al. [41] compared conventional composites with nanocomposites and found that microleakage values for groups bonded with nanocomposite were significantly higher. Arhun et al. [42] compared the amount of microleakage in Transbond XT under ceramic and metal orthodontic brackets. The metal brackets showed significantly more microleakage than the ceramic brackets between the bracket-adhesive interfaces. This may be caused by incomplete photopolymerization through the metal bracket and the difference in the thermal expansion of materials. Metal brackets expand and contract more than ceramic ones, which can lead to microleakage. Trulock™ Light Activated Adhesive composite material had a statistically significantly higher occurrence of microleakage compared to other composites. The occurrence of microleakage in this material has not been tested yet, thus we could not compare our results with previous studies.

In this study, we detected that the microleakage occur more frequently between the enamel and adhesive than between the adhesive materials and the base of the orthodontic bracket. This has also been confirmed by other authors [8, 36, 37]. In contrast, Atash et al. [38] found a higher amount of microleakage at the “occlusal bracket-adhesive” interface.

In all the materials tested, we found that a higher amount of microleakage occurred in the gingival region, which is consistent with other studies [8, 36, 43, 44]. This finding could be attributed to the anatomy of the tooth surface curvature, which may result in a thicker layer of adhesive at the gingival side [8, 42]. Also, the curing method itself can be another contributing factor as the photopolymerization light is typically applied to the occlusal side during treatment [44]. Several authors assessed microleakage under orthodontic brackets using different curing techniques [42, 43, 45, 46]. Davari et al. [46] compared the use of LED unit and plasma arc curing in both metal and ceramic orthodontic brackets. LED unit caused more microleakage than plasma arc curing. This study also showed less microleakage in ceramic brackets. The same results claimed Arhun et al. [42]. Ulker et al. [43] compared microleakage development under orthodontic brackets bonded with high-intensity light curing lights and conventional halogen lights but did not find a significant difference between them.

In vitro studies, including the dye penetration method, are commonly used to detect microleakage under orthodontic brackets [8, 38, 40, 47]. The 2% aqueous solution of methylene blue has been widely used by other authors of similar studies [38, 47]. In contrast, Alkis et al. and Ulker et al. [8, 43] used a 0.5% solution of basic fuchsine. The dying process was performed for 24 h in all presented studies. Another method for testing of microleakage is the use of micro-CT. We could not use this method while testing metal orthodontic brackets because of the artifacts and the inability to properly analyse the microleakage development. Another reason why we did not use micro-CT is that very small microleakage could not be detected with this method.

There are several limitations that must be considered when conducting this in vitro study. Oral environment is constantly subjected to thermal fluctuations and mechanical forces. Thermal changes caused by hot and cold dishes and beverages lead to the expansion and contraction of materials in the mouth. The expansion and contraction by thermal stimuli that repeat many times per day affect the stress of materials present in the oral cavity [24]. Although the thermocycling protocol was established, it still differs a lot from the oral mouth environment. Changes in pH and mechanical loading were not performed. Further, interindividual variability of the oral environment of every patient under the in vitro conditions cannot be simulated.

## Conclusions

This study concluded that microleakage exhibited by adhesive materials can differ significantly. According to our findings, the most promising composite materials for practical use in orthodontics are GC Ortho Connect, Transbond XT, and Light Bond, as they exhibited the lowest amount of microleakage after photopolymerization. The highest amount of microleakage was detected in GCF and this property should be taken into consideration when this material is applied. A higher microleakage occurred in the gingival margin and careful polymerization of the material in this region is thus recommended. Additional investigation into the curing methods, the shear bond strength and adhesive remnant index of these materials when using ceramic and metal orthodontic brackets is necessary. In addition, further in vivo examination of materials used for bonding orthodontic brackets and their interactions with mechanical and chemical processes in the oral cavity should be performed.

## Data Availability

The data that support the findings of this study are available on request from the corresponding author.

## Abbreviations

°C: Degree Celsius
AICc: Akaike’s information criterion
CI: Confidence interval
GC: GC Fuji Ortho LC
GCO: GC Ortho Connect
LB: Light Bond
min: Minute
mm: Millimetre
PDP: Proportional dye penetration
s: Second
TB: Transbond XT
TL: Trulock™ Light Activated Adhesive
